# Amygdala 5-HTT Gene Network Moderates the Effects of Postnatal Adversity on Attention Problems: Anatomo-Functional Correlation and Epigenetic Changes

**DOI:** 10.3389/fnins.2020.00198

**Published:** 2020-03-17

**Authors:** Randriely Merscher Sobreira de Lima, Barbara Barth, Danusa Mar Arcego, Euclides José de Mendonça Filho, Andrew Clappison, Sachin Patel, Zihan Wang, Irina Pokhvisneva, Roberto Britto Sassi, Geoffrey B. C. Hall, Michael S. Kobor, Kieran J. O'Donnell, Ana Paula Santana de Vasconcellos Bittencourt, Michael J. Meaney, Carla Dalmaz, Patrícia Pelufo Silveira

**Affiliations:** ^1^Programa de Pós-Graduação em Neurociências, Instituto de Ciências Básicas da Saúde (ICBS), Universidade Federal do Rio Grande do Sul, Porto Alegre, Brazil; ^2^Integrated Program in Neuroscience (IPN), McGill University, Montreal, QC, Canada; ^3^Department of Psychiatry, Faculty of Medicine, McGill University, Montreal, QC, Canada; ^4^Programa de Pós-Graduação em Psicologia, Instituto de Psicologia, Universidade Federal do Rio Grande do Sul, Porto Alegre, Brazil; ^5^Ludmer Centre for Neuroinformatics and Mental Health, Douglas Hospital Research Centre, McGill University, Montreal, QC, Canada; ^6^Mood Disorders Program, Department of Psychiatry & Behavioural Neurosciences, McMaster University, Hamilton, ON, Canada; ^7^Department of Psychology, Neuroscience & Behaviour, McMaster University, Hamilton, ON, Canada; ^8^Centre for Molecular Medicine and Therapeutics, BC Children's Hospital Research Institute, Department of Medical Genetics, The University of British Columbia, Vancouver, BC, Canada; ^9^Departamento de Ciências Fisiológicas, Universidade Federal do Espírito Santo, Vitoria, Brazil; ^10^Singapore Institute for Clinical Sciences, Agency for Science, Technology and Research (A*STAR), Singapore, Singapore

**Keywords:** early environment, serotonin transporter, ADHD, DNA methylation, ePRS, parallel independent component analysis, attention, impulsivity

## Abstract

Variations in serotoninergic signaling have been related to behavioral outcomes. Alterations in the genome, such as DNA methylation and histone modifications, are affected by serotonin neurotransmission. The amygdala is an important brain region involved in emotional responses and impulsivity, which receives serotoninergic input. In addition, studies suggest that the serotonin transporter gene network may interact with the environment and influence the risk for psychiatric disorders. We propose to investigate whether/how interactions between the exposure to early life adversity and serotonin transporter gene network in the amygdala associate with behavioral disorders. We constructed a co-expression-based polygenic risk score (ePRS) reflecting variations in the function of the serotonin transporter gene network in the amygdala and investigated its interaction with postnatal adversity on attention problems in two independent cohorts from Canada and Singapore. We also described how interactions between ePRS-5-HTT and postnatal adversity exposure predict brain gray matter density and variation in DNA methylation across the genome. We observed that the expression-based polygenic risk score, reflecting the function of the amygdala 5-HTT gene network, interacts with postnatal adversity, to predict attention and hyperactivity problems across both cohorts. Also, both postnatal adversity score and amygdala ePRS-5-HTT score, as well as their interaction, were observed to be associated with variation in DNA methylation across the genome. Variations in gray matter density in brain regions linked to attentional processes were also correlated to our ePRS score. These results confirm that the amygdala 5-HTT gene network is strongly associated with ADHD-related behaviors, brain cortical density, and epigenetic changes in the context of adversity in young children.

## Introduction

Attention Deficit Hyperactivity Disorder (ADHD) is a prevalent psychiatric condition characterized by symptoms of inattention, impulsivity, and hyperactivity (American Psychiatric Association, 2013). ADHD is a developmental disorder with symptoms often evident before 6 years of age (Faraone et al., 2003). Both genetic and environmental factors make a substantial contribution to the etiology of ADHD (Matthews et al., 2014). Childhood adversities are associated with the development of ADHD (Boecker et al., 2014; Björkenstam et al., 2017; Bock et al., 2017) while candidate gene studies suggest that low serotonergic activity is associated with increased impulsive-aggressive behavior (Faraone et al., 2003). Alterations in serotonin synthesis, breakdown and transport have been associated to ADHD-related phenotypes (Oades, 2010).

Serotonin or 5-hydroxytryptamine belongs to the indolamine family of neurotransmitters, and influences physiological processes, including autonomic function, motor activity, hormone secretion, and behavioral processes such as cognition, emotion, reward and attention (Carlsson, 1987; Greengard, 2001). The synthesis of 5-HT occurs in serotonergic neurons (Strüder and Weicker, 2001) where 5-HT is stored into presynaptic vesicles by a vesicular monoamine transporter (Sakowski et al., 2006). After being released, 5-HT is recycled through a process of active re-uptake by the serotonin transporter (5-HTT) (Hoffman et al., 1998). The 5-HTT gene, also known as *SLC6A4*, is localized in the long arm of chromosome 17. This gene encodes an integral membrane protein that transports the neurotransmitter serotonin from synaptic spaces into presynaptic neurons, and represents a primary mechanism for the regulation of serotonergic activity (Heils et al., 2002). 5-HTT is expressed in brain regions implicated in attention and memory such as the amygdala, hippocampus, and prefrontal cortex (Frankle et al., 2004; Oquendo et al., 2007; Puig and Gulledge, 2011). The amygdala is predominantly modulated by the 5-HT system, and plays an important role in attentional processes (Pessoa, 2011). Several studies have observed that variations specifically in this gene are associated with impulsivity, hyperactivity, and ADHD (Halperin et al., 1997; Manor et al., 2001; Seeger et al., 2001). Likewise, Genetic variation in *SLC6A4* also associates with individual differences in DNA methylation, a functional epigenetic modification of DNA, and *SLC6A4* gene expression. Interestingly, such genetic effects on DNA methylation emerged as a function of exposure to adversity early in life (Beach et al., 2010; Vijayendran et al., 2012); highlighting the importance of the interplay between the genome and the environment in the regulation of *SLC6A4* gene expression.

A large number of studies focuses on heritability and candidate genes involved in ADHD. However, dysfunctions usually involve several molecular pathways that may result from variants in many genes, each of them contributing with only weak effects to the phenotype (Gaiteri et al., 2014). Analyzing the effect of genes co-expressed with a gene of interest allows the integration of information on gene networks and the functional relationships between these genes (Silveira et al., 2017; Miguel et al., 2019). Multivariate approaches like parallel ICA (p-ICA) are used to identify relationships between clusters of functionally related SNPs that are statistically correlated and phenotype components, such as brain structure (Pearlson et al., 2015; Khadka et al., 2016). Studies have been using p-ICA to correlate biological pathways with variations in gray matter density, which represents an important tool to identify an anatomical-functional basis for a behavioral phenotype (Khadka et al., 2016; Miguel et al., 2019). Additionally, stressors in early life are known to alter the expression of genes and cellular processes that influence behavior, and one of the molecular processes that contributes to this change is DNA methylation (Vinkers et al., 2015; Hing et al., 2018). However, the mechanisms linking these associations need to be further investigated.

Based on this evidence, we hypothesized that early life adversity would interact with individual differences in genetic variation within the *SLC6A4* gene network to predict attention-related problems in childhood. We constructed an expression-based polygenic risk score (ePRS) that reflects the function of the amygdala 5-HTT gene network, and described how this polygenic predictor interacted with postnatal adversity to predict attention/hyperactivity and brain gray matter density. We also investigated the association between postnatal adversity and the ePRS of the 5-HTT gene network on variations in DNA methylation across the genome.

## Methods and Materials

### Participants

We used data from two prospective birth cohorts based in Canada (Maternal Adversity, Vulnerability and Neurodevelopment—MAVAN) and in Singapore (Growing Up in Singapore Toward Healthy Outcomes—GUSTO), to analyze the gene network by environment interaction effects on behavioral outcomes.

The study sample MAVAN is a birth cohort that followed up children from birth up to 6 years of age in Montreal (Quebec), and Hamilton (Ontario), Canada (O'Donnell et al., 2014). Mothers aged 18 years or above, with singleton pregnancies, and fluent in French or English were included in the study. Severe maternal chronic illness, placenta previa, and history of incompetent cervix, impending delivery, or a fetus/infant affected by a major anomaly or gestational age <37 weeks were the exclusion criteria. Approval for the MAVAN project was obtained by the ethics committees and university affiliates (McGill University and Université de Montréal, the Royal Victoria Hospital, Jewish General Hospital, Centre hospitalier de l'Université de Montréal and Hôpital Maisonneuve-Rosemount) and St. Joseph's Hospital and McMaster University, Hamilton, Ontario, Canada. Informed consent was obtained from all participants. In GUSTO, the replication cohort, pregnant women aged 18 years and above were recruited at the National University Hospital (NUH) and KK Women's and Children's Hospital (KKH), being of Chinese, Malay or Indian ethnicity with homogeneous parental ethnic background. Mothers receiving chemotherapy, psychotropic drugs or who had type I diabetes mellitus were excluded. Informed written consent was obtained from each participant (Soh et al., 2014).

### Genotyping

Genome-wide platforms (PsychArray/PsychChip, Illumina) were used to genotype 242,211 autosomal SNPs of buccal epithelial cells of children in MAVAN, according to manufacturer's guidelines. SNPs with a low call rate (<95%), low *p*-values on Hardy-Weinberg Equilibrium (HWE) exact test (*p* <1e-40), and minor allele frequency (MAF <5%) were removed. Afterwards, imputation using the Sanger Imputation Service was performed and SNPs with an info score >0.80 were retained for the analysis, resulting in 20,790,893 SNPs (McCarthy et al., 2017).

In GUSTO, genotying was performed using Infinium OmniExpressExome array and split by ethnicity for quality checks. Non-autosomal SNPs, SNPs with call rates <95%, minor allele frequencies <5%, and failed Hardy-Weinberg equilibrium *p*-value of 10–6 were removed. Variants discordant with their respective subpopulation in the 1,000 G reference panel were removed (Chinese: EAS with a threshold of 0.20; Malays: EAS with a threshold of 0.30; Indian: SAS with a threshold of 0.20). Samples with call rate <99%, cryptic relatedness and sex/ ethnic discrepancies were excluded. The resulting data were pre-phased using SHAPEIT v2.837 with family trio information. We then used Sanger Imputation Service for imputation, choosing 1,000 G Phase 3 as reference panel and imputed “with PBWT, no pre-phasing” as the pipeline. Imputed data that were non-monomorphic, had biallelic SNPs and an INFO score >0.80 were retained. Imputed genotyping data that were common in all three ethnicities (5,771,259 SNPs) were used for further analyses.

The population structure of the MAVAN and GUSTO cohorts were evaluated using principal component analysis of all autosomal SNPs that passed the quality control without low allele frequency (MAF>5%) and not in high linkage disequilibrium (*r*^2^ >0.2) across 500 kb regions (Price et al., 2006; Wray et al., 2014). Based on the inspection of the scree plot, the first three principal components were the most informative of population structure in both cohorts and were included in all subsequent analyses.

### DNA Methylation Analyses

DNA methylation analyses were done in buccal epithelial cells (Catch-All Swabs, Epicenter, USA) in MAVAN children at a mean age of 6.99 years. Details of DNA extraction, preprocessing and quality control procedures were described in Garg et al. (2018). Briefly, genomic DNA (750 ng) was bisulfite converted using the EZ-DNA Methylation Kit (Zymo Research, USA) and hybridized on Infinium HumanMethylation450 beadchip array (450 K, Illumina). Data was processed in R using the Minfi package (Aryee et al., 2014). Samples that failed standard Minfi quality control procedure were removed. All remaining samples had a high call rate (>95%). Probes with a low call rate (<75%), a high detection *p*-value (*p* > 0.05) and a low number of beads (<3 in >5% of the cohort) were also removed. We also used ComBat (Johnson et al., 2007) to iteratively adjust our data for unwanted technical variation associated with experimental batch, array row (sentrix row), and plate position (sample column). We also predicted buccal epithelial cell content of each sample (Smith et al., 2015) and included the proportion of buccal cells per sample as a covariate in all models describing variation in DNA methylation levels. A full explanation of the DNA methylation analyses is provided in Garg et al. (2018).

### 5-HTT Co-expressed Genes and ePRS

**Figure 1:**
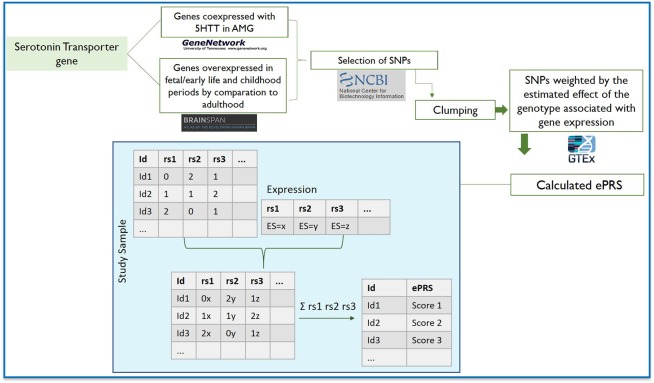
Flowchart depicting the steps involved in creating the expression-based polygenic risk score based on genes co-expressed with serotonin transporter gene in amygdala using gene co-expression databases: GeneNetwork was used to generate a co-expression matrix with 5-HTT gene in the amygdala in mice (absolute value of the co-expression correlation *r* ≥ 0.5); BrainSpan was then used to identify consensus human transcripts from this list; BrainSpan was also used for selecting genes differentially expressed at ≥1.5-fold during child and fetal development as compared to adulthood within the same brain areas. Based on their functional annotation in the National Center for Biotechnology Information, U.S. National Library of Medicine, we gathered all the existing SNPs from these genes and subjected this list of SNPs to linkage disequilibrium clumping. We used a count function of the number of alleles at a given SNP (rs1, rs2…) weighted by the estimated effect of the genotype associated with gene expression. The sum of these values from the total number of SNPs provides the amygdala 5-HTT ePRS score.

The expression-based polygenic risk score was created considering genes co-expressed with the serotonin transporter gene (5-HTT-ePRS) in amygdala, according to the protocol previously described by Silveira et al. (2017) and Miguel et al. (2019). The genetic score from the 5-HTT gene network was created using: GeneNetwork (http://genenetwork.org), BrainSpan (http://www.brainspan.org), NCBI Variation Viewer (https://www.ncbi.nlm.nih.gov/variation/view), and The Genotype-Tissue Expression (GTEx) (https://gtexportal.org/home/) (Lonsdale et al., 2013). GeneNetwork was used to generate a list of genes coexpressed with 5-HTT in the amygdala in mice (Mulligan et al., 2016). Only genes with absolute value of co-expression correlation higher or equal to 0.5 were retained. The gene list generated by GeneNetwork was then filtered using BrainSpan to identify consensus transcripts enriched in the fetal and childhood human brain (Miller et al., 2014). Since we were interested in genes that were active during early developmental periods, we selected autosomal transcripts expressed in the amygdala at least 1.5-fold more during fetal and child development (all fetal samples and up to the first 5 years of age) as compared to adult samples. Based on their functional annotation in the National Center for Biotechnology Information, U.S. National Library of Medicine (NCBI Variation Viewer), using GRCh37.p13, we gathered all the SNPs from these genes and merged this list with the SNPs from the GTEx data in human amygdala to form a list of common SNPs. The list of common SNPs was then subjected to linkage disequilibrium clumping (*r*^2^ < 0.25), which resulted in 463 SNPs. In ePRS calculation, alleles at a given cis-SNP were weighed by the estimated effect of the genotype on gene expression (expression quantitative trait loci from GTeX, in which the effect allele is the alternative allele). Final ePRS was obtained by summation over all SNPs accounting for the sign of correlation coefficient between the genes and 5HTT gene expression. For more information on ePRS calculation, see Hari Dass et al. (2019). The summation of these values from the total number of SNPs provides the amygdala 5-HTT-ePRS score (Table 1 shows the final gene list). Figure 1 illustrates the steps involved in the creation of the ePRS.

**Table 1:** Genes selected for composing the genetic score (5-HTT-ePRS).

**Gene**	**Ensembl**	**Description**
ADAMTS7	ENSG00000136378	ADAM metallopeptidase with thrombospondin type 1 motif, 7
ATAD2B	ENSG00000119778	Atpase family, AAA domain containing 2B
AURKA	ENSG00000087586	Aurora kinase A
CHD7	ENSG00000171316	Chromodomain helicase DNA binding protein 7
CHRNB4	ENSG00000117971	Cholinergic receptor, nicotinic, beta 4
CNGA3	ENSG00000144191	Cyclic nucleotide gated channel alpha 3
COL3A1	ENSG00000168542	Collagen, type III, alpha 1
DLL3	ENSG00000090932	Delta-like 3 (Drosophila)
DNMT3B	ENSG00000088305	DNA (cytosine-5-)-methyltransferase 3 beta
EHMT2	ENSG00000204371	Euchromatic histone-lysine N-methyltransferase 2
EIF4EBP1	ENSG00000187840	Eukaryotic translation initiation factor 4E binding protein 1
ELN	ENSG00000049540	Elastin
GAS5	ENSG00000234741	Growth arrest-specific 5 (non-protein coding)
GTSE1	ENSG00000075218	G-2 and S-phase expressed 1
HMGB1	ENSG00000189403	High mobility group box 1
HNRNPA1	ENSG00000135486	Heterogeneous nuclear ribonucleoprotein A1
LRRC26	ENSG00000184709	Leucine rich repeat containing 26
NBEAL1	ENSG00000144426	Neurobeachin-like 1
NHLH1	ENSG00000171786	Nescient helix loop helix 1
PKN1	ENSG00000123143	Protein kinase N1
PRKDC	ENSG00000253729	Protein kinase, DNA-activated, catalytic polypeptide
PTK7	ENSG00000112655	PTK7 protein tyrosine kinase 7
RAD54L	ENSG00000085999	RAD54-like (*S. cerevisiae*)
RBM12B	ENSG00000183808	RNA binding motif protein 12B
RBM4	ENSG00000173933	RNA binding motif protein 4
RPL27A	ENSG00000166441	Ribosomal protein l27a
RPL36	ENSG00000130255	Ribosomal protein L36
SCLT1	ENSG00000151466	Sodium channel and clathrin linker 1
SFRP1	ENSG00000104332	Secreted frizzled-related protein 1
TEAD2	ENSG00000074219	TEA domain family member 2
TRIM58	ENSG00000162722	Tripartite motif containing 58
TSKU	ENSG00000182704	Tsukushi small leucine rich proteoglycan homolog (*Xenopus laevis*)
USP49	ENSG00000164663	Ubiquitin specific peptidase 49
WDR62	ENSG00000075702	WD repeat domain 62
ZCCHC7	ENSG00000147905	Zinc finger, CCHC domain containing 7

### Postnatal Adversity Score

The postnatal adversity score was created combining multiple indicators of adversity. The instruments included as indicators of postnatal adversity were the following: (1) *The Health and well-being questionnaire*: short versions of multiple measures (Kramer et al., 2001): (a) Presence of chronic disease during pregnancy or severe acute conditions; (b) A subscale from the Daily Hassles was used to measure how often, and to what degree, the woman had lacked money for basic needs since the beginning of pregnancy (Kanner et al., 1981); (c) The Marital Strain Scale of Pearlin and Schooler was used to assess chronic stress with the romantic partner (Pearlin and Schooler, 1978); (d) The Abuse Assessment Screen was used to assess conjugal violence (Newberger et al., 1992; Parker et al., 1993); (e) Questions about anxiety during pregnancy (Lobel et al., 1992; Parker et al., 1993). (2) *Smoking during pregnancy*: composed of yes or no questions. (3) *Household gross income* (Daveluy et al., 1998). (4) *Depression questionnaire* (Centre of Epidemiological Studies) (CES-D): a 20 Likert-Scale item instrument applied to assess the symptoms of depression (Radolf, 1977). (5) *Child Health Questionnaire*: Includes questions on acute, chronic conditions, and hospitalizations (Plante et al., 2002). (6) *Maternal mental health*: Beck Depression Inventory, a 21-question self-report questionnaire (Beck et al., 1961); Edinburgh Postnatal Depression Scale (EPDS), a 10-item self-report scale designed to screen for postpartum depression (Cox et al., 1987); State-Trait Anxiety Inventory (STAI), a self-report instrument consisted of two forms of 20 items each to measure psychic components of state and trait anxiety (Spielberger, 2010). (7) *Attachment*: The Preschool Separation—Reunion Procedure (PSRP) was applied at 36 months and used to measure attachment security in preschool-aged children. The task consists of a baseline interaction, followed by two separation and reunion episodes lasting 5 min; scoring was based on video coding (reliability *k* = 0.83). Four categories were assessed: secure, ambivalent, avoidant and disorganized (Cassidy and Marvin, 1992). (8) *Family Assessment Device*: 60-item self-report instrument developed to assess the six dimensions of the family functioning outlined in the McMaster Model of Family Functioning (Moss et al., 2004). A general functioning scale assesses overall health-pathology (Kabacoff et al., 1990). In GUSTO the indicators of adversity were similar, except that there was no information on attachment styles in this cohort. For each continuous variable we applied 15th or 85th percentile cut-off to categorize subjects in exposed or not to that event. The total score represents the summation of all points. The earliest postnatal time point available for each variable was used for the calculation of the adversity score.

### Behavioral Outcome: Child Behavior Checklist

The Child Behavior Checklist (CBCL) questionnaire which includes 100 items was used to evaluate emotional, behavioral, and social difficulties in preschool children. The focus of this study was in scales related with attention and hyperactivity problems, so we chose ADHD Problems, Attention Problems and Externalizing Problem scales for this work. An externalizing scale is computed by summing scores on items related to attention problems and aggressive behaviors (Achenbach, 2011).

A total of 137 children from the MAVAN cohort and 401 from the GUSTO cohort had complete data (genotype, information on early life environment, and CBCL scores) and were included in the study.

### Gray Matter Density

Structural MRI of the whole brain was acquired using a 3T trio Siemens scanner available at Cerebral Imaging Center, Douglas Research Centre (Montreal, Canada) (1 mm isotropic 3D MPRAGE, sagittal acquisition, 256 × 256 mm grid, TR= 2,300 ms, TE = 4 ms, FA = 9°) and a GE MR750 Discovery 3T MRI scanner at the Imaging Research Centre, St. Joseph's Healthcare (Hamilton, Canada) (3D inversion recovery-prepped, T1-weighted anatomical data set, fSPGR, axial acquisition, TE/TR/flip angle = 3.22/10.308/9, 512 × 512 matrix with 1 mm slice thickness and 24 cm FOV. T1-weighted images were processed by computational Anatomy Toolbox (CAT12) from the Statistical Parametric Mapping software (SPM12). In the preprocessing step, the images were normalized and segmented into gray matter and white matter. After a high-dimensional Diffeomorphic Anatomical Registration Through Exponentiated Lie Algebra (DARTEL) normalization, a smoothing process was applied using 8 mm full width half maximum kernel.

### Enrichment Analysis

The gene network data were retrieved from GeneMANIA (Warde-Farley et al., 2010) (https://genemania.org) database and the gene-gene interaction networks were constructed and visualized in the Cytoscape software (Saito et al., 2012). Enrichment analysis was performed using MetaCore™ (Clarivate Analytics). Data for analysis of the co-expression data during development was extracted from BrainSpan (Miller et al., 2014). Epigenes (genes and proteins involved in epigenetic regulation) within the networks were investigated using the Epifactors Database (http://epifactors.autosome.ru/) (Medvedeva et al., 2015). Localization of epigenes on cell classes in the brain were made using cell types enrichment (https://www.brainrnaseq.org/) (Zhang et al., 2014). Enrichment analysis for molecular function, biological process, protein class, cell components and pathways were performed in Panther (http://pantherdb.org/) (Mi et al., 2019).

### Statistical Analysis

Data was analyzed using R (https://www.rstudio.com/) (R Foundation for Statistical Computing, 2018). Significance levels for all measures were set at α < 0.05. Linear regression analysis was applied to examine the effects of interaction between the polygenic score and the adversity score on the behavioral outcomes (CBCL), adjusting for sex and population stratification. For the analyses with a significant interaction term, simple slope analyses were conducted to describe the differences. Population stratification was composed by models adjusted by principal components (Patterson et al., 2006; Price et al., 2006). For that, population structure was analyzed to identify the presence of a systematic difference in allele frequencies between subpopulations in a population. We added the principal components to adjust for false results due to ancestry differences. We pruned our datasets to common variants (MAF>0.05) that were not in linkage disequilibrium (*r*^2^ < 0.20) with a sliding window (50 kilobases) approach that examined linkage disequilibrium in increments of 5 SNPs using PLINK 1.9 (Price et al., 2006). Principal component analysis was performed using SMARTPCA on this pruned dataset and generated a scree plot (see Hari Dass et al., 2019, for scree plot for the MAVAN cohort). Based on the inspection of the scree plot, the first three principal components were the most informative of population structure in both cohorts and were included in all analyses.

A multivariate approach called parallel independent component analysis (pICA) was applied to identify relationships between two different data modalities focusing on inter-related patterns in a data driven manner (Price et al., 2006). The goal of this method is to discover independent components from these two data modalities, in addition to the relationship between them (Liu and Calhoun, 2014). The pICA is a variant of ICA for multimodality processing that extracts maximally independent components within each data modality separately, while also maximizing the association between modalities using an entropy term based on information theory, thus enhancing the interconnection by maximizing the linkage function in a joint estimation process (Liu and Calhoun, 2014; Pearlson et al., 2015).

Using this approach we sought to find the relationship between the SNP-based ePRS-5-HTT (or genotype x GTEx gene expression slope at each SNP comprised by the ePRS-5-HTT) and the voxel-based gray matter in the whole brain, instead of investigating the relationship between the crude genotype and the gray matter-voxel-based measures. The groups for comparison (25 children with high adversity score and 24 children with low adversity score) were defined by the postnatal environment aggregated with population stratification (ethnicity) for adjustment. The number of independent components estimated using minimum description length criteria (Calhoun et al., 2001) was 17 for genetic data and 8 for MRI data. Loading coefficients, which describe the presence of the identified component across participants (Liu et al., 2012) were extracted for each component, modality, and participant. The mean participant-specific loading coefficients of these components between children from high and low-adversity-score groups was compared using Student's *t*-test.

Further we explored if variation in the levels of DNA methylation was predicted by postnatal adversity score, ePRS-5-HTT, or their interaction. We used the following models of linear regression analyses to test these associations:

1. CpG ~ sex + PC1 + PC2 + PC3 + A postnatal
2. CpG ~ sex + PC1 + PC2 + PC3 + ePRS
3. CpG ~ sex + PC1 + PC2 + PC3 + A_ postnatal * ePRS

CpG represents methylation levels at a single variably methylated probe; sex is the biological sex of participant; PC(1, 2, or 3) represents the population stratification principal components from the genetic data, and A_ postnatal represents the postnatal adversity score.

For each variably methylated CpG we compared the Akaike information criterion (AIC) across the three models to identify the model that best explained variation in DNA methylation at a given CpG. We utilized AIC since it permits the comparison of non-nested models.

## Results

Baseline comparisons between low and high ePRS groups (median split) were done in the two cohorts. Differences in means on the main confounding variables were tested using Student's *t*-test for independent samples and Chi-square Test was applied for categorical variables. No differences were found in relation to the main confounding variables in MAVAN (Table 2). In GUSTO (Table 2), we observed a higher prevalence of breastfeeding for at least 3 months in the low ePRS group (66%) vs. high ePRS group (56%). To confirm our results we adjusted the interaction by breastfeeding in GUSTO. The same results were observed.

**Table 2:** Description of the baseline characteristics of the MAVAN and GUSTO samples according to high and low amygdala ePRS-5-HTT.

**Sample description**	**Total (*n* = 145)**	**Low 5-HTT (*n* = 69)**	**High 5-HTT (*n* = 76)**	***p*-value**
**MAVAN**				
Sex—male	49.7% (72)	53.6% (37)	46.1% (35)	0.36
Maternal age at birth (years)	30.60 (4.62)	30.20 (4.58)	30.95 (4.65)	0.33
Gestational age (weeks)	39.18 (1.21)	39.25 (1.25)	39.12 (1.17)	0.52
Birth weight (grams)	3,308 (452)	3,380 (477)	3,242 (420)	0.06
Breastfeeding (months)	7.53 (4.80)	7.54 (4.61)	7.52 (4.99)	0.98
Smoking during pregnancy	12.4% (18)	10.1% (7)	14.5% (11)	0.43
Maternal education—University degree or above	57.9% (84)	62.3% (43)	53.9% (41)	0.30
Low family income	15.2% (20)	15.4% (10)	14.9% (10)	0.94
**Sample description**	**Total (*****n*** **=** **401)**	**Low 5-HTT (*****n*** **=** **197)**	**High 5-HTT (*****n*** **=** **204)**	***p*****-value**
**GUSTO**				
Sex - male	51.9% (208)	50.3% (99)	53.4% (109)	0.52
Maternal age at birth (years)	31.21 (5.12)	31.33 (5.27)	31.1 (4.98)	0.65
Gestational age (weeks)	38.48 (1.27)	38.43 (1.36)	38.53 (1.18)	0.39
Birth weight (grams)	3,139 (425)	3,119 (420)	3,158 (430)	0.36
Breastfeeding for at least 3 months	61.0% (241)	66.1% (127)	56.2% (114)	0.042^*^
Smoking during pregnancy	2.0% (8)	1.0% (2)	2.9% (6)	0.16
Maternal education—University degree or above	35.4% (142)	39.1% (77)	31.9% (65)	0.13
Household income < SG$2,000	13.5% (54)	11.2% (22)	15.7% (32)	0.18

*Numbers are presented as mean (SD) or percentage (number of participants)*.

* *Significant differences between low and high ePRS groups (p <0.05)*.

### Interaction Between ePRS-5-HTT in the Amygdala and Postnatal Adversity Score Moderates the Behavior in Children

**Figure 2:**
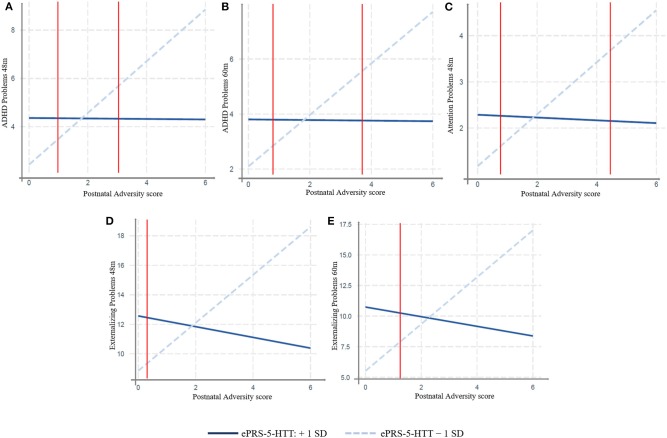
Interaction between Postnatal Adversity Score and amygdala-based ePRS-5-HTT on: **(A)** ADHD problems 48 and **(B)** 60 months (*N* = 137), **(C)** Attention Problems at 48 months **(D)** Externalizing Problem at 48 months **(E)** and 60 months. Increased postnatal adversity exposure is associated with more problematic behavior outcomes as the ePRS decreases. MAVAN cohort, *N* = 125–137.

In MAVAN children, we found a significant effect of interaction between the adversity score and the amygdala-based ePRS-5-HTT on domains of the CBCL related to attentional problems and hyperactivity: ADHD Problems at 48 months (β = –71.19, *p* = 0.0009) and 60 months (β = –62.32, *p* = 0.006); Attention Problems at 48 months (β = –39.07, *p* = 0.010) and a trend at 60 months (β = –27.81, *p* = 0.07); and Externalizing Problem at 48 months (β = –131.06, *p* = 0.03) and 60 months (β = –151.64, *p* = 0.01). Simple slope analysis showed that increased postnatal adversity exposure is associated with more ADHD problems (48 months: β = 0.93, *p* = 0.0001; 60 months: β = 1.07, *p* < 0.0001), attentional problems (β = 0.56, *p* = 0.0004); externalizing problems (48 months: β = 1.62, *p* = 0.01; 60 months: β = 1.91, *p* = 0.002), as the ePRS-5-HTT score decreases (Figure 2). After correction for multiple testing using Bonferroni-Holm method, ADHD Problems at 48 and 60 months were still significant.

**Figure 3:**
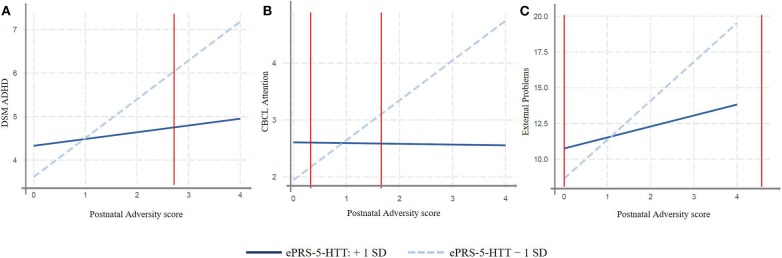
Interaction effect between Postnatal Adversity Score and amygdala-based ePRS-5-HTT on: **(A)** ADHD problems at 4 years, **(B)** attention problems at 4 years, and **(C)** Externalizing Problems at 4 years. Increased postnatal adversity exposure is associated with outcomes related to more Behavioral problems as ePRS decreases. GUSTO cohort, *N* = 401.

We replicated our findings in a different population. More specifically, we analyzed whether the interaction between the adversity score and the amygdala-based ePRS-5-HTT would predict attention problems in children in the GUSTO cohort, and similar results were found. We observed significant effect of interactions between adversity score and the amygdala-based ePRS-5-HTT on CBCL scales: ADHD problems at 4 years (β = −32.79, *p* = 0.02) and attention problems at 4 years (β = −31.77, *p* = 0.0014) and externalizing problems at 4 years (β = –87.06, *p* = 0.031; Table 3). A simple slope analysis showed that increased postnatal adversity exposure is associated with more ADHD problems (β = 0.89, *p* < 0.0001), as well as with higher attentional (β = 0.70, *p* < 0.0001), and externalizing problems (β = 2.72, *p* < 0.0001) as the ePRS-5-HTT score decreases (Figure 3). After correction for multiple testing, all the associations remained significant.

**Table 3:** Results of interactions between the adversity score and the amygdala-based ePRS-5-HTT on CBCL behavior of children in MAVAN and GUSTO cohorts; and evidence of differential susceptibility.

**Interactions between the adversity score and the amygdala-based ePRS-5-HTT**				
**MAVAN**			**GUSTO**	
**Outcome**	**Adversity** **×** **ePRS**		**Outcome**	**Adversity** **×** **ePRS**
Attention problems 48 m	β = −39.07, *p =* 0.010^*^		Attention Problems 48m	*β = –*31.77, *p =* 0.0014^*^
ADHD problems 48 m	*β = –*71.19, *p =* 0.0009^*^		ADHD Problems 48m	*β =* −32.79, *p =* 0.02^*^
Externalizing problems 48 m	*β = –*131.06, *p =* 0.03^*^		Externalizing Problems 48m	*β =* −87.06, *p =* 0.03^*^
Attention problems 60 m	*β =* −27.81, *p =* 0.07			
ADHD problems 60 m	*β =* −62.32, *p =* 0.006^*^			
Externalizing problems 60 m	*β = –*151.64, *p =* 0.01^*^			
**Evidence of differential susceptibility**				
**Outcome**	**Cohort**	**PoI**	**PA**	**Differential susceptibility**
Attention problems 48 m	MAVAN	0.17	0.60	Yes
ADHD problems 48 m	MAVAN	0.15	0.60	Yes
ADHD problems 60 m	MAVAN	0.15	0.61	Yes
Externalizing problems 48 m	MAVAN	0.17	0.60	No
Externalizing problems 60 m	MAVAN	0.27	0.80	No
Attention problems 48 m	GUSTO	0.85	0.43	Yes
ADHD problems 48 m	GUSTO	0.09	0.43	No
Externalization problems 48 m	GUSTO	0.12	0.78	Yes

*PoI and PA values close to 0.50 suggest strong evidence for differential susceptibility*.

* *Significant interactions between the adversity score and the amygdala-based ePRS-5-HTT (p < 0.05)*.

We also analyzed whether these interactions were consistent with the differential susceptibility model (Roisman et al., 2012). We verified if regions of significance were inside the possible range of value for the environmental score, calculated proportion of interaction (PoI) and proportion affected (PA). Table 3 shows these results in MAVAN and GUSTO cohorts. In every case we find some evidence (PoI and PA values close to 0.50 suggest strong evidence for differential susceptibility) for differential susceptibility. All three criteria showed that interactions were consistent with differential susceptibility. These results suggest that the same genetic background associated with the 5-HTT gene network is affected by the exposure to adversity but also more protected in supportive environments with regards to ADHD and Attention Problems outcomes. Pol and PA are consistent with differential susceptibility in MAVAN and GUSTO cohort (see Table 3).

These results suggest a presence of a strong genome x environment interaction effect on behavior outcomes in children. Exposure to postnatal adversity and decreased ePRS-5-HTT are associated with negative outcomes in CBLC at different ages in different cohorts. This suggests that the amygdala 5-HTT ePRS is a strong predictor of attention-related processes in the context of variation in the environmental quality in line with the differential susceptibility paradigm.

### Postnatal Adversity, ePRS-5-HTT, and Their Interaction Contribute to DNA Methylation Variations

**Figure 4:**
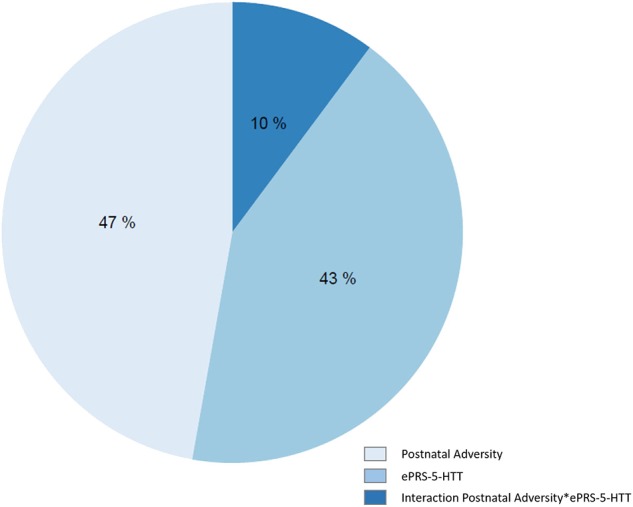
Variations in DNA methylation levels were predicted by postnatal adversity score, ePRS-5-HTT, and the interaction between postnatal adversity score and ePRS-5-HTT. Each variable on chart denotes one model. AIC was used to compare three models for each CpG and select the best one. Percentages on chart represent the number of CpGs that are best explained (lowest AIC) by a specific model. All models were adjusted for population stratification and sex.

We identified a total of 54,295 variably methylated probes cross the genome. For each probe we compared AIC across three models to explore the prediction of the DNA methylation by postnatal adversity, ePRS or their interaction. We observed that postnatal adversity explained DNA methylation levels better than other predictors for 47% of all variably methylated sites. The ePRS-5-HTT main effect was a better predictor for 43% of the variably methylated sites and the interaction between the two factors—for 10% of CpGs (Figure 4).

### Enrichment Analysis of the 5-HTT Co-expression Networks

**Figure 5:**
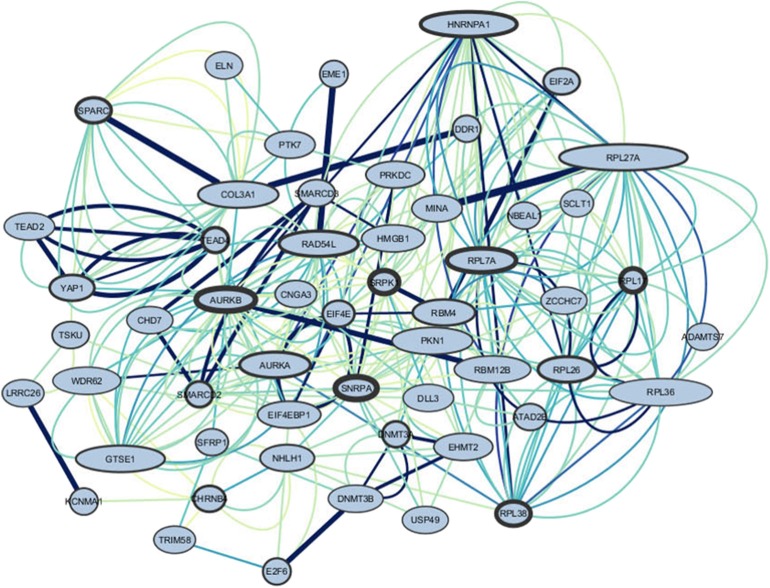
Genes interactions of the amygdala 5-HTT co-expression network. The border of the genes represents the out-degree of these nodes, meaning the number of outgoing relationships. The larger the border, the stronger the relationship of the gene to other genes. The size of the nodes represents the in-degree, i.e., the number of incoming relationships with neighbors. The bigger the node, the higher the relationships of other genes to the target gene. The edges represent co-expression. Color and thickness were used to identify the most co-expressed genes, darker, and thicker represent higher co-expression.

We performed enrichment analysis on the genes selected for composing the genetic score (complete list of genes on Table 1). The relationship between genes was performed using GeneMANIA, and the interaction networks were constructed in the Cytoscape software. Figure 5 shows the gene interactions. Using Cytoscape, we calculated the degrees and betweenness of the 5-HTT network. Nodes above the threshold (mean + 1 SD) were considered central nodes (high betweenness indicated bottlenecks and high degrees indicated hubs) (Neves de Oliveira et al., 2018). The two most important hubs and bottlenecks of this list included: HNRNPA1 (Heterogeneous Nuclear Ribonucleoprotein A1), a protein coding gene, member of a family of ubiquitously expressed heterogeneous nuclear ribonucleoproteins, which are RNA-binding proteins that associate with pre-mRNAs in the nucleus and influence pre-mRNA processing, as well as other aspects of mRNA metabolism and transport; and PRKDC, a protein kinase, which encodes the catalytic subunit of the DNA-dependent protein kinase. AURKA (Aurora kinase) is the most import hub gene of the gene network co-expressed with 5-HTT in amygdala. The protein encoded by this gene is a cell cycle-regulated kinase that appears to be involved in microtubule formation and/or stabilization at the spindle pole during chromosome segregation. The most important bottleneck is RAD54L. The protein encoded by this gene is involved in homologous recombination and repair of DNA (Stelzer et al., 2016).

**Figure 6:**
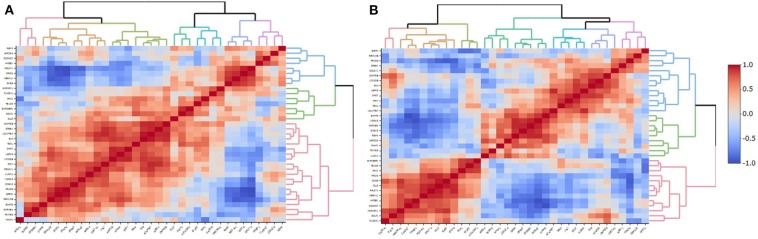
Co-expression of the genes included in the ePRS-5-HTT in the amygdala in the **(A)** infancy and early childhood, and **(B)** adulthood periods in humans. Each vertical line represents correlation with a unique gene. Genes from the same expression quantification tend to cluster together and could be visualized in red (positive correlation), or blue (negative correlation) patterns. Infancy and early childhood period ranges from 0 to 4 years of age (*N* = 7); Adulthood ranges from 20 to 40 years (*N* = 6). Data for this analysis was extracted from BrainSpan.

BrainSpan data was used to correlate the expression levels of the genes included in the ePRS-5-HTT in the amygdala in two periods (a) infancy and early childhood, and (b) adulthood. We observed large clusters of highly co-expressed genes in both periods (Figure 6). However, different gene clusters were observed during infancy/early childhood and during adulthood, suggesting that there is a developmental influence on the design of the clusters of co-expression of these genes.

**Figure 7:**
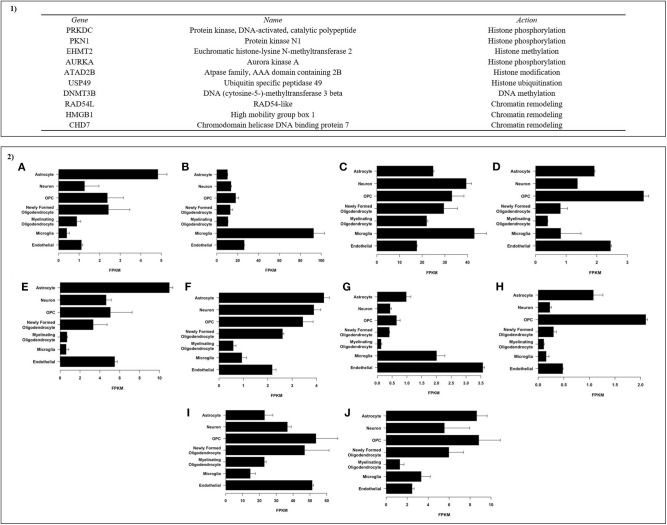
Epifactors included in the expression-based polygenic risk score of the 5-HTT in the amygdala: **(1)** Genes classified as epifactors, involved in histone phosphorylation and methylation, chromatin remodeling, and DNA methylation; **(2)** Cell classes in the brain where these epifactors are expressed. Each graphic represents a gene: **(A)** PRKDC, **(B)** PKN1, **(C)** EHMT2, **(D)** AURKA, **(E)** ATAD2B, **(F)** USP49, **(G)** DNMT3B, **(H)** RAD54L, **(I)** HMGB1, and **(J)** CHD7.

**Figure 8:**
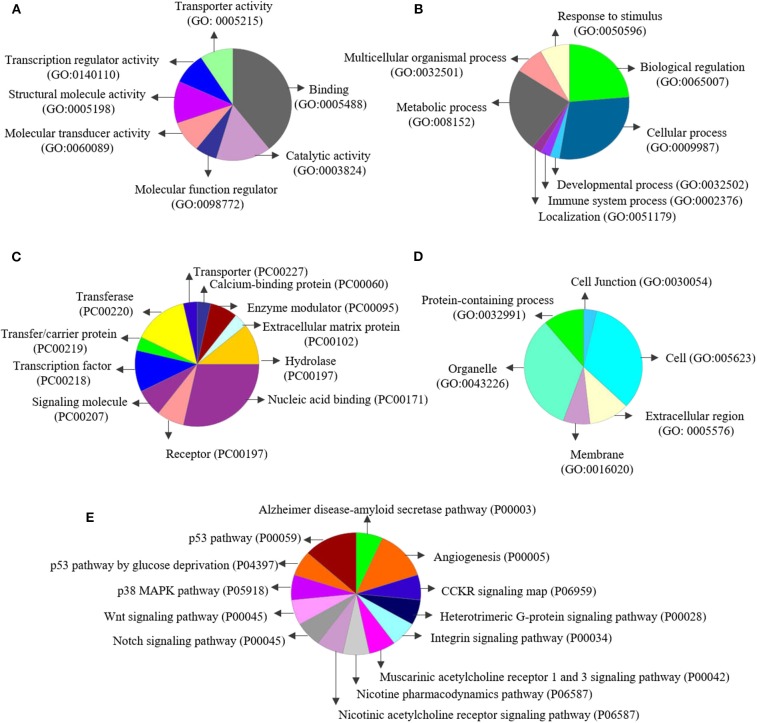
Enrichment analysis performed using Panther (http://pantherdb.org/): Enrichment for **(A)** molecular functions, **(B)** biological processes, **(C)** protein classes, **(D)** cell components, and **(E)** pathways.

Enrichment analyses performed in Metacore® shows two important pathways related with DNA damage and epigenetic regulation of gene expression (see all results in Table 4). The enrichment analysis for gene ontology shows enrichment processes related to chromosome organization, DNA conformational changes, DNA methylation; and nervous system development (such as generation of neurons and developmental process). Interestingly, of the 35 genes on the list of genes co-expressed with 5-HTT in the amygdala, 10 are classified as epifactors and are involved in histone phosphorylation and methylation, chromatin remodeling and DNA methylation (Figure 7). We also analyzed the cell classes in the brain where these epifactors are more expressed, and most of them are localized in astrocytes, neurons, and oligodendrocyte precursor cells (Figure 7). Enrichment analyses performed in Panther shows molecular functions related with transporter activity and binding, biological process related with developmental processes, cellular processes, and metabolic processes. At same time, proteins classes were enriched for transporter, nucleic acid binding, and transferases. Finally, this gene network was enriched in pathways related to muscarinic acetylcholine and nicotine receptors signaling pathways (Figure 8).

**Table 4:** Pathway Maps and Gene Ontology Processes related to genes included in the expression-based polygenic risk score of the 5-HTT in the amygdala.

**Enrichment by Metacore^®^**		
**Maps**	***p*****-value**	**FDR**
**Pathway Maps**		
Apoptosis and survival_nAChR in apoptosis inhibition and cell cycle progression	0.0009	0.046
DNA damage_Mismatch repair	0.001	0.046
Transcription_Role of heterochromatin protein 1 (HP1) family in transcriptional silencing	0.0017	0.046
Transcription_Sin3 and NuRD in transcription regulation	0.0017	0.046
Canonical Notch signaling pathway in colorectal cancer	0.0023	0.046
Signal transduction_Adenosine A2A receptor signaling pathway	0.003	0.046
Translation_Translation regulation by Alpha-1 adrenergic receptors	0.003	0.046
TLRs-mediated IFN-alpha production by plasmacytoid dendritic cells in SLE	0.003	0.046
Transcription_Epigenetic regulation of gene expression	0.003	0.048
**GO Processes**		
Chromosome organization	0.00000004	0.00005
DNA geometric change	0.0000001	0.00008
Nervous system development	0.0000001	0.00009
Chromatin organization	0.0000002	0.00010
Generation of neurons	0.0000004	0.00016
Cellular response to stress	0.0000005	0.00016
System development	0.0000005	0.00016
Neurogenesis	0.000001	0.00023
DNA conformation change	0.000002	0.00027
Developmental process	0.000017	0.00109
DNA methylation on cytosine within a CG sequence	0.00004	0.00162
Positive regulation of DNA ligation	0.00004	0.00162

### SNP-Based ePRS-5-HTT and the Voxel-Based Gray Matter Density

**Figure 9:**
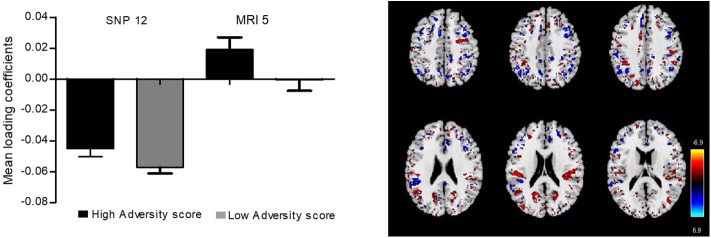
A bar plot of the mean loading coefficients of brain magnetic resonance imaging (MRI) component and genetic component. Student's *t*-test was performed to compare loading coefficients means between groups of high and low adversity score for SNPs (single nucleotide polymorphisms) and MRI.

In order to perform an anatomical-functional correlation of our findings, we analyzed the relationship between brain gray matter density and the SNPs used to create the ePRS-5-HTT in 49 children from MAVAN that had MRI and genotype data available. The Parallel ICA identified seven significant relationships between regional gray matter volume and SNP-based ePRS. The most significant relationships between regional gray matter density and SNP-based ePRS-5-HTT data was on the genetic component 12 and MRI component 5 (*r* =0.704, *p* = 1.62e-08). The comparison of mean loading coefficients of these components between children from high- and low adversity score groups by Student's *t*-test indicated no statistically significant differences (Figure 9), suggesting that SNPs on the ePRS-5-HTT list moderate gray matter density in specific brain regions, but this effect is not moderated by the adversity experienced on these parameters.

To define the significant SNPs in each component, we used a threshold of higher than 2.5 and lower than 2.5 (Z-Threshold>±2.5). In component 12, we found 14 significant SNPs, and the enrichment analysis by Metacore® demonstrated that these SNPs are involved especially in regulation of neuronal migration [false discovery rate (FDR) = 0.0082], cerebral cortex development (FDR = 0.02), neurogenesis (FDR = 0.025), regulation of cell migration (FDR = 0.030), and cell development (FDR = 0.04). This group of SNPs were related to gray matter density located mainly in cortical areas, such as superior, middle and inferior frontal gyrus, temporal gyrus, precuneus, and inferior parietal lobule (MRI component 5). All the significant brain regions and SNPs are listed in Tables 5, 6.

**Table 5:** Significant brain regions associated with gray matter density and SNP-based ePRS-5-HTT data on MRI component 5 by Parallel ICA.

**Brain-phenotype component 5**			
**Area**	**Brodmann area**	**L/R volume (cc)**	**Random effects: Max value (*****x, y, z*****)**
**POSITIVE**			
Middle temporal gyrus	19	0.4/0.6	5.1 (−53, −53, −9)/5.2 (62, −28, −10)
Sub-gyral	*	0.4/0.4	5.0 (−34, −40, 38)/6.0 (45, −47, −9)
Precuneus	7, 31	0.4/0.2	5.0 (−22, −61, 38)/4.0 (9, −53, 44)
Middle frontal gyrus	6, 8, 10	0.3/0.6	3.9 (−22, 22, 38)/4.7 (18, −11, 61)
Inferior temporal gyrus	19, 20, 21, 37	0.3/0.4	4.9 (−34, −3, −36)/5.3 (49, −57, −2)
Postcentral gyrus	3, 4	0.3/0.4	4.7 (−50, −19, 20)/4.2 (55, −20, 37)
Fusiform gyrus	19, 20, 37	0.3/0.3	5.5 (−53, −11, −23)/4.3 (48, −49, −13)
Superior frontal gyrus	6, 10	0.3/0.3	4.8 (−24, 51, −5)/4.5 (18, −11, 66)
Cerebellar declive	*	0.3/0.2	4.9 (−37, −71, −22)/5.6 (30, −77, −20)
Lingual gyrus	18	0.3/0.2	4.5 (−12, −77, 0)/4.0 (27, −64, −6)
Superior temporal gyrus	42	0.3/0.1	4.3 (−55, −34, 16)/3.8 (61, −34, 18)
Middle occipital gyrus	*	0.2/0.3	5.4 (−22, −92, 3)/5.2 (42, −70, −7)
Supramarginal gyrus	40	0.2/0.0	3.9 (−45, −50, 25)/−999.0 (0, 0, 0)
Precentral gyrus	6	0.1/0.3	4.1 (−49, 8, 8)/5.1 (33, −19, 48)
Medial frontal gyrus	10, 11	0.1/0.3	3.7 (−7, 39, 30)/4.2 (10, 51, 2)
**NEGATIVE**			
Middle frontal gyrus	6, 9, 10, 11, 46	0.8/1.2	4.8 (−30, 8, 52)/5.5 (36, 18, 39)
Inferior parietal lobule	39, 40	0.6/0.6	5.5 (−48, −47, 42)/5.4 (31, −53, 45)
Inferior frontal gyrus	9, 44, 45, 47	0.6/0.6	4.9 (−48, 32, 0)/5.0 (46, 4, 30)
Sub-gyral	*	0.5/0.4	5.4 (−31, −51, 38)/4.8 (25, −35, 52)
Superior temporal gyrus	13, 22, 39	0.4/0.2	5.1 (−58, −40, 19)/3.8 (64, −26, 10)
Medial frontal gyrus	6, 10, 11	0.4/0.1	5.2 (−13, 4, 59)/4.1 (16, 62, −4)
Precuneus	7	0.3/0.6	4.3 (−4, −61, 31)/4.2 (13, −48, 55)
Superior frontal gyrus	8, 9, 11	0.3/0.5	4.6 (−34, 46, 16)/5.1 (9, 58, 22)
Precentral gyrus	9, 44	0.3/0.1	6.5 (−48, 19, 9)/4.4 (39, 8, 37)
Postcentral gyrus	2, 3	0.3/0.1	5.0 (−25, −34, 53)/4.6 (27, −31, 55)
Supramarginal gyrus	40	0.2/0.3	4.0 (−50, −48, 33)/4.3 (55, −48, 30)
Middle temporal gyrus	37	0.2/0.2	4.0 (−50, −65, 6)/5.0 (49, −51, 7)
Superior parietal lobule	7	0.2/0.1	3.9 (−40, −56, 48)/4.1 (31, −56, 48)

* *Does not have a close match to a particular brodmann area*.

**Table 6:** Dominant SNPs (Z-Threshold = 2.5) associated with gray matter density on SNP component 12 by Parallel ICA.

**Gene**	**SNP**	**Z Score**
TRIM58	rs61857833	8.86
TRIM58	rs9787332	6.85
TRIM58	rs55634584	6.65
ZCCHC7	rs68046074	5.24
TRIM58	rs34268703	4.87
WDR62	rs117559678	−4.84
TRIM58	rs2101702	−3.42
CNGA3	rs4851129	−3.30
COL3A1	rs3134656	3.03
WDR62	rs139494942	2.77
GTSE1	rs79334853	2.74
TRIM58	rs4925574	2.64
GTSE1	rs3817874	2.53
TRIM58	rs112538341	2.53

## Discussion

The main aim of this study was to analyze whether a genetic score based on genes co-expressed with the serotonin transporter in the amygdala moderates the impact of adversity exposure in early life on behaviors related to attention and hyperactivity, as well as comorbidities associated with this condition. We demonstrated that the expression-based polygenic risk score reflecting the function of an amygdala 5-HTT gene network interacts with postnatal adversity exposure, influencing attention and hyperactivity problems in different cohorts and different ages. We found a correlation between the postnatal adversity score, amygdala ePRS-5-HTT scores and their interaction on variations of DNA methylation across the genome. Additionally, we observed that SNPs used to create the ePRS-5-HTT are positively and negatively correlated with variations in gray matter density. These results together confirm that the gene network co-expressed with 5-HTT is strongly associated with behavioral, brain cortical density, and epigenetic changes in the context of adversity, that strongly correlate with their impact on attentional problems in young children.

The attention deficit hyperactivity disorder is characterized by symptoms of inattention, impulsivity, and hyperactivity. Often, ADHD is associated with emotions and behavior dysfunctions, such as affect dysregulation, irritability, and mood (Matthews et al., 2014). We demonstrated that early life adversity is strongly associated not only with attention and hyperactivity problems, but also with behaviors correlated with ADHD, such as externalizing behaviors and total problems (as measured through the CBCL). Externalizing behavioral problems reflect the child negatively acting on the external environment and consist of disruptive, hyperactive, and aggressive behaviors (Nolan et al., 1996; Liu, 2004). Our finding corroborates the associations between ADHD problems and comorbidities, primarily related to externalizing behavior problems.

The serotonergic system is associated with several behavioral processes, in domains such as cognition, emotion, reward, and attention (Carlsson, 1987; Greengard, 2001). Variations in the 5-HTT gene have been reported to be associated with impulsivity, hyperactivity, and ADHD (Halperin et al., 1997; Manor et al., 2001; Seeger et al., 2001; Heils et al., 2002). The amygdala is a complex structure rich in serotonergic terminals, and it is involved in several processes related to emotion, mechanisms of vigilance, and attention (Pessoa, 2011). While several studies have focused on the effects of serotonergic function on comorbidities, our approach presents a biologically defined gene network of genes co-expressed with the serotonin transporter in the amygdala. We demonstrated that variation across the 5HTT gene network moderated the effects of postnatal adversity on attention and hyperactivity problems. Furthermore, we observed that the interaction between genetic variation and environment on attention and hyperactivity problems provided support for the differential susceptibility hypothesis. These results suggest that individuals whose behavior is negatively affected by environmental adversity could be the same individuals to benefit most from an enriched/supportive environment (Belsky et al., 2009). Belsky et al. have suggested that children more sensitive to context might be more affected by developmental factors, being that an exposure to adversity or to more enriched environments in early life (Belsky, 1997). These results provide evidence that the 5-HTT gene network is associated with vulnerability and resilience to environmental variations, which can have implications for both preventive and therapeutic interventions.

Our findings showed that the postnatal adversity score and gene network of 5-HTT in the amygdala predict variation in DNA methylation across the genome. In fact, several studies have demonstrated that trauma in early life affects DNA methylation (Beach et al., 2010; Ouellet-Morin et al., 2013). Infant attachment is also associated with variations in genome-wide DNA methylation (Garg et al., 2018) and the early social environment has persisting influence until adult life on the DNA methylation variation across the genome (O'Donnell et al., 2018). The serotoninergic system, especially the serotonin transporter, regulates a number of epigenetic modifications (Ismaylova et al., 2018). However, we show that a broader analysis of 5-HTT gene network variation is associated with epigenetic changes. Interestingly, several genes included in the 5-HTT gene network are associated with epigenetic processes. As shown in Figure 7, 10 of the 35 genes in the amygdala 5HTT gene network are associated with histone phosphorylation and methylation, chromatin remodeling, and DNA methylation. This explains the strong association between the ePRS and variations of DNA methylation observed in our model. Although the ePRS-5-HTT in the amygdala and postnatal adversity explain most of the variations observed, it is important to emphasize that the interaction between these two factors also predicts variations in DNA methylation across the genome. These results may explain, at least in part, the mechanism by which adversity exerts its long-term effects, especially in attention hyperactivity problems.

We also analyzed the relationship between the SNPs used to create the ePRS-5-HTT in amygdala and brain gray matter density, in order to obtain anatomical-functional resolution in our findings. We observed an association between these SNPs and gray matter density in cortical areas involved in memory, attention, information processing, and decision-making, such as precuneus, superior and inferior temporal gyrus, frontal gyrus, and inferior parietal lobule, respectively (Shapiro et al., 2002; du Boisgueheneuc et al., 2006; Wallentin et al., 2006; Vickery and Jiang, 2009; Tops and Boksem, 2011). Clinical studies have been demonstrated that smaller volumes of frontal, parietal and occipital cortices were associated with the higher rates of ADHD (Botellero et al., 2017; Suffren et al., 2017). In fact, ADHD is a disorder associated with cognitive control and reward response. Theories that address ADHD as a disorder of executive control have considered atypical activity or connectivity specifically in prefrontal regions and posterior cortical regions (Miller and Cohen, 2001; Aron et al., 2004; Matthews et al., 2014). At the same time, theories that consider ADHD as a sensory and reward dysfunction have associated this disorder with brain regions involved in motivation, reward and emotional regulation, and with dysregulation of emotional control (Matthews et al., 2014). Even though we analyzed brain gray matter density, not activity or connectivity, it is interesting that the results pointed to an association between SNPs of genes co-expressed with 5-HTT in amygdala and gray matter density in cortical regions associated with executive control, suggesting that both theories may be important for typical behaviors observed in this disorder, and that this gene network could have an important role in this moderations.

These results together point to an association between genes and the environment: the gene network co-expressed with 5-HTT in the amygdala was consistently associated with behavioral outcomes, variations of gray matter density and DNA methylation. In fact, the biological processes enriched for this gene network show a high association with nervous system development and epigenetic pathways. Previous studies have shown that ADHD patients with higher *SLC6A4* promoter methylation status had significantly worse hyperactive-impulsive symptoms, suggesting the potential role of epigenetics pathways in attention problems (Park et al., 2015). Furthermore, epigenes of this gene network were enriched in different cells, such as astrocytes, neurons and precursors of oligodendrocytes, which demonstrates the wide distribution of these factors. Interestingly, this gene network was also enriched for pathways of the cholinergic system, which have been associated with a number of cognitive functions, including memory, attention, and emotional processing (Sarter et al., 2001, 2006; Bentley et al., 2003). The presence of these pathways demonstrates that complex dysfunctions are probably associated not only with a gene, but possibly with a network of genes, and several systems, cells and brain regions (Gaiteri et al., 2014). Exposure to stress has been shown to affect methylation of Lys4 in histone 3 (Han et al., 2012). Methylation of this amino acid residue has been reported to affect the activity of DNMT3B (Liang et al., 2002; Chen et al., 2003) one of the enzymes coded by a gene in the ePRS-5-HTT network. This is a possible mechanism through which early adversity could epigenetically influence the function of this network and, thus, interact with our genetic score.

In conclusion, our present findings provide support for the impact of exposure to postnatal adversity on attention deficit hyperactivity disorder and externalizing problems, showing that the 5-HTT gene network is an important moderator of these effects. Our data also supports the hypothesis that this gene network has important impact in brain regions related with attention and cognitive processes. Additionally, the 5-HTT gene network and postnatal adversity are associated with variation in DNA methylation, which may explain the mechanism of long-term effects of postnatal adversity. Our study extends the knowledge of how exposure to postnatal adversity affects behavior and highlights the importance of analyzing not just a candidate gene in psychiatric disorders, but an entire gene network.

## Data Availability Statement

The raw data supporting the conclusions of this article will be made available by the authors, without undue reservation, to any qualified researcher upon reasonable request.

## Ethics Statement

The studies involving human participants were reviewed and approved by MAVAN: (McGill University and Université de Montréal, the Royal Victoria Hospital, Jewish General Hospital, Centre Hospitalier de l'Université de Montréal and Hôpital Maisonneuve-Rosemount) and St. Joseph's Hospital and McMaster University, Hamilton, Ontario, Canada. GUSTO: National Healthcare Group Domain Specific Review Board and the Sing Health Centralized Institutional Review Board. Written informed consent to participate in this study was provided by the participants' legal guardian/next of kin.

## Author Contributions

RL, PS, and CD designed the experiments. RS, GH, MK, KO'D, PS, and MM performed the data collection. CD and AB performed the enrichment analysis. SP performed the ePRS. ZW, AC, IP, and EM performed statistical analysis. BB, DA, CD, and PS analyze the results and wrote the manuscript with RL.

### Conflict of Interest

The authors declare that the research was conducted in the absence of any commercial or financial relationships that could be construed as a potential conflict of interest.
